# Cultural Perspectives, Feelings and Coping Behaviors during the COVID-19 Pandemic: A Case Study of Romanian Students

**DOI:** 10.3390/ijerph191912445

**Published:** 2022-09-29

**Authors:** Simona Șimon, Marcela Alina Fărcașiu, Gabriel-Mugurel Dragomir

**Affiliations:** Faculty of Communication Sciences, Politehnica University of Timișoara, 300006 Timișoara, Romania

**Keywords:** coping behavior, COVID-19 pandemic, cultural perspectives, face masks, Romanian students, socio-cultural patterns, feelings, civism, interpersonal relationships

## Abstract

Socio-cultural patterns and communication styles differ from culture to culture. As such, the way in which people deal with a crisis situation is also culture-dependent. The COVID-19 pandemic has pointed, once more, to the cultural diversity of the world through a variety of reactions to the measures imposed by the global spread of the deadly virus. The present research aims at identifying the feelings, coping behaviors and communication patterns of the younger Romanian generation during the COVID-19 pandemic and at explaining them from a cultural standpoint, in an effort to raise awareness of the cultural (un)predictability of human reactions to certain external stimuli. The survey conducted online on 409 students at Politehnica University of Timișoara (Romania) revealed that most of students’ socio-cultural behavior could have been anticipated by the Romanian authorities when they decided a certain pandemic action plan, and that the few unexpected results indicate the versatility of a culture that is still changing under the Western European influence caused by the fact that Romania has adhered to European Union principles for more than fifteen years.

## 1. Introduction

The COVID-19 outbreak in China, in December 2019, and the spread of the novel deadly virus worldwide restructured contemporary society, having long-term consequences difficult to predict. On 11 March 2020, the World Health Organization publicly acknowledged that the coronavirus outbreak could be characterized as a global pandemic [1]. Five days later, on 16 March 2020, in an effort to contain the surge in infections, in Romania, a state of emergency was declared, which imposed a national lockdown, followed, in May, by a state of alert [2,3,4] that continued until March 2022 [5]. The preventive and protective measures taken by the official bodies against the spread of the SARS-CoV-2 virus led to restrictions on people’s movements, and on the ways in which businesses and society as a whole operated. The period of uncertainty, frustration, fear, mask-wearing, extreme hygiene, social distancing and isolation that followed made people find new and, sometimes, even creative and culture-bound solutions [6,7,8,9,10,11,12,13] to communicate both verbally and nonverbally, in an attempt to better cope with the psychological pressure [6,7,8,9,10,11,12,13,14,15,16,17]. In this context, collectivistic cultures have reacted better to the COVID-19 pandemic than individualistic ones, the former being able to embrace the new normal and the vaccine against the SARS-CoV-2 virus more easily, and thus to save lives [8,9,12]. Interestingly, during the COVID-19 pandemic, representatives of collectivistic cultures faced life-threatening situations light-heartedly, being more optimistic and more psychologically balanced than their individualistic counterparts [11,12]. While individualistic cultures have believed in COVID-19 conspiracy theories, and have felt powerless and less willing to comply with the safety measurements, collectivistic ones have accepted social distancing as well as hygiene-related measures more openly and have not felt that powerless [12,13]. In other words, in crisis situations, “collectivism encourages a powerful response, but individualism removes a sense of power and replaces it with potentially harmful conspiracy beliefs” [13] (p. 671).

Among the important causes enhancing the mental health of the population in pandemic times, religion has been singled out [10]. This, however, has also had a negative effect on the spread of the virus, since it has favored large gatherings of people and, as such, it has eased the transmission of the virus. For example, one of the major causes that turned a Romanian Roma village into an “epidemiological hotspot” [7] was their paying their last respects, in large numbers, to a deceased Roma villager. Consequently, a deeper acceptance, by religious devotees, of safety measurements as well as of scientific proofs and realities might be essential in stemming the spread of the SARS-CoV-2 virus in some communities [10]. 

Interestingly, within the same society, behaviors may differ so that minority cultural groups as well as different age groups may behave differently from the dominant cultural group existing in that society, and, as such, customized pandemic measures should be taken into account by governing bodies in order to succeed in combating the disease [6,12]. Considering this, the present article builds on the survey conducted among students at Politehnica University of Timișoara (Romania) in order to identify their feelings during the COVID-19 pandemic, and their coping behavior to adjust to the new normal, i.e., the new life they had to live in order to protect themselves and others from infectious disease. To achieve this goal, four research objectives were set, namely to identify the respondents’ feelings about the first year of the pandemic, to identify the respondents’ preferences for a certain design or color of face masks worn during the COVID-19 pandemic, to identify the way in which the respondents communicated interpersonally with friends, family members or colleagues during the COVID-19 pandemic, and, finally, to identify the degree of civism indicated by the respondents’ reactions to the citizens who were not wearing a face mask when they met. The results were interpreted in relation to some cultural patterns usually associated with Romania (cf. Section 2 of this article), raising awareness of the (un)predictability of the younger generation’s reactions to stressful situations, on the one hand, and, on the other, of the culture-bound behavior of the population, an aspect that, in our view and that of others [18,19], should be considered more often by authorities whenever they take major decisions that impact people’s lives.

## 2. Literature Review

### 2.1. Socio-Cultural Patterns and Communication Styles across the World 

The way in which people react to the positive as well as to the negative factors that may affect their daily activities is determined by the culture that has shaped their personalities since human behavior, be it verbal or nonverbal, and is eminently culture-bound [13,20,21,22,23,24,25,26,27,28,29,30,31,32,33,34]. Thus, culture, broadly defined as “the collective programming of the mind that distinguishes the members of one group or category of people from others” [34] (p. 3), influences distinctively the way in which humans communicate, as E.T. Hall [31] emphasized in the previous century. He distinguished between high-context and low-context communication or message; the first one is indirect, implicit and ambiguous, while the second one is direct, explicit and clear. In order to decipher the message transmitted in a high-context culture, the participants in the communication process resort to the cultural values and common knowledge they share, since communication is highly contextualized. In a low-context culture, however, the message is encoded in the discourse, i.e., there is no need to go beyond the uttered words to understand what is meant by that specific piece of discourse. The Arab, Japanese, Chinese, Korean, Vietnamese, Romanian, Middle Eastern, Mediterranean and Latin American cultures are viewed as high-context ones, while the North American, German, British, Swiss and other Northern European cultures as low-context ones [30,31,32,35]. 

Communication is, however, achieved not only through linguistic means, but also through non-linguistic ones such as colors and touch patterns [26,36,37,38,39,40]. Colors are “a universal language” [26] (p. 51), conveying different meanings in different cultures. Consequently, there are colors associated with royalty, faithfulness, jealousy, strength, melancholy and happiness, to mention but a few, that vary with culture as well as a cultural preference for warmer or cooler colors. Warmer colors are preferred in countries with a warm climate, whereas cooler colors in those with a cold climate. Furthermore, cultures also vary in degree of interpersonal closeness, as Hall [40] highlighted. He differentiated between high-contact cultures and low-contact ones, in other words between cultures characterized by a high sensory stimulation and a low sensory stimulation, respectively. Knowing cultural contact patterns is essential in avoiding misunderstandings and leading a fulfilling life in a globalized society. Hence, high-contact cultures are the South American, Southern and Eastern European as well as the Arab ones, while low-contact cultures are North European and Asian ones [41]. Interestingly, North American and Australian cultures are considered moderate in their contact patterns [37]. To put it differently, “cultures in cooler climates tend to be more task-oriented and interpersonally ‘cool’, whereas cultures in warmer climates tend to be more interpersonally oriented and interpersonally ‘warm’” [42] (p. 75).

Other dimensions that distinguish between cultures were singled out in 1980 by G. Hofstede [43] and further developed in 2010 by G. Hofstede, G.J. Hofstede and M. Minkov [44], in 2011 by G. Hofstede [34] as well as in 2018 by S. Beugelsdijk and C. Welzel [45]. The aforementioned scholars describe cultures in terms of power distance versus closeness, uncertainty avoidance versus acceptance, individualism versus collectivism, masculinity versus femininity, long-term orientation versus short-term orientation, indulgence versus restraint. Power distance versus closeness shows people’s attitude towards hierarchy and authority, and the acceptance or rejection of social inequalities. For example, power distance is reflected in the following situations: power does not need to be legitimized since it has always existed in society before things could be labeled as good or bad; hierarchy is accepted presupposing unequal existential roles; subordinates follow the superior’s indications, without expecting to be consulted; governments are autocratic and changed during riots; corruption is the rule and scandals are overseen; income is distributed unevenly in society. Closeness, on the other hand, is mirrored by the following aspects: power should be used legitimately in the case of something bad happening; hierarchy presupposes unequal roles established for convenience purposes, and therefore subordinates expect their opinion to be asked for; governments are pluralist ones, elected by the people and changed without much fuss; corruption is not the rule, and any scandal may cause the end of a political career; income is distributed quite evenly in society. Thus, Asian, African, Latin and East European cultures are high-distance ones, while Germanic and English-speaking cultures are low-distance ones. 

Uncertainty avoidance versus acceptance is about people’s capacity to adapt to new situations, the degree of stress that people feel when they have to face uncertainty and their preference to work and live in a well-structured or less structured society. Hence, uncertainty avoidance is shown by the following: uncertainty is considered to be a threat; high degree of stress, anxiety, neuroticism and emotionality; subjective health and well-being scores are low; intolerance to new and different ideas and/or persons; clarity and structure are needed in all situations; people need rules, even if they do not obey them; people feel small and incompetent in relation to the authorities and the other way around; people believe in religious, philosophical and scientific ultimate truths and grand theories. Acceptance, though, is quite the opposite: uncertainty is accepted and dealt with when it comes; people are easy-going, relaxed, self-controlled, less anxious than their counterparts in the uncertainty avoidance cultures; tolerance to as well as curiosity about new and different ideas and/or persons; people know how to handle chaos and ambiguity, without feeling uncomfortable; rules are not preferred; people and authorities are equally important and competent; relativism and empiricism are embraced in religion, philosophy and science. Uncertainty avoidance is high in Latin, East and Central European, Germanic and Japanese cultures and lower in Nordic, Anglo-Saxon and Chinese cultures.

Individualism versus collectivism points to the entire social construction that relies either on the individual and their immediate family or on a cohesive in-group and an extensive family. In other words, individualism is characterized by the following: I-consciousness; each individual is perceived as a unique individual; privacy is valued; expressing one’s opinion is expected; completing a task is more important that preserving a relationship; the pronoun I is often used in discourses. Collectivism is described by: WE-consciousness; a strong sense of belonging and loyalty to a group, therefore individuals are assessed from the perspective of their membership of in-groups and out-groups; pre-determined opinions formulated by in-groups; feelings of shame when norms are transgressed; harmonious interactions; relationships prevalent over tasks; avoidance of the use of the pronoun I in discourses. Since individualism tends to develop the more developed the society, it is a characteristic of Western cultures. Eastern countries are usually less developed economically and, as such, eminently collectivist. Interesting, however, is the fact that Japan is somehow in-between these two cultural dimensions, displaying features of both of them. Furthermore, individualism and collectivism have an effect on peoples’ creativity, manifested in their private and professional lives [39], as individuals from individualist cultures tend to be more creative than those from collectivist cultures. This happens because, for the latter, usefulness and fear of failure are of paramount importance, and for the former, novelty and uniqueness. Since one cannot innovate without taking certain risks, it is understandable why, usually, Western individualist cultures are more prone to creative thinking and innovation in all fields of activity than Eastern collectivist cultures that tend to always take the safe path.

Masculinity versus femininity illustrates the gender-specific values and attitudes characterizing a society as a whole. Thus, masculinity stands for assertiveness and competitivity, while femininity is associated with modesty and attentiveness. It is either the masculine or the feminine pattern that tends to determine the behavior both of men and of women in a certain society. Some aspects reveal a masculine society: there is a maximum gender role differentiation; assertiveness and ambition are highly appreciated; work is more important than family; the strong ones are admired. A feminine society is indicated by the opposite: there is a minimum gender role differentiation; modesty and attentiveness are highly valued; work is as important as family; the weak ones are sympathized with. Therefore, Japanese, Germanic and some Latin cultures such as the Mexican and Italian ones are considered highly masculine, while Dutch and Nordic cultures are highly feminine. Mixed features of masculinity and femininity are found in some Latin cultures such as the French, Spanish, Portuguese and Chilean ones as well as in some Asian cultures such as the Korean and Thai ones.

Long-term orientation versus short-term orientation points to people’s perceptions of the past, the present and the future and the manner in which these interrelate and influence each other, thus determining people’s course of action and behavior. In long-term orientation cultures, the best is yet to come, people’s adaptability to their circumstances is praised, traditions are subject to change, tasks are shared among family members, eagerness to learn from other people is a defining trait, people are thrifty, perseverant and save money for future investment, and hard work is key to success. In short-term orientation cultures, the past and the present weigh heavier than the future, people never change, people are omniscient as regards their ability in assessing the good and evil in everything, traditions are sacred and static, imperatives define family life, one’s country is the only one that is praised and admired, helping others is essential, and luck influences success. The aforementioned studies [34,43,44,45] highlight that the long-term oriented societies are the East Asian, the Eastern and Central European ones as opposed to American, Latin American, Australian, African and Muslim societies that are rather short-term oriented. Medium-term orientation is spotted in South-Asian, South and North European societies.

Finally, the last dimension that characterizes a culture is indulgence versus restraint, i.e., flexible social norms that allow people to enjoy life and have fun versus strict social norms regulating people’s behavior and encouraging them to control their most human desires. Thus, in societies characterized by indulgence, people are happy and perceive themselves as being in control of their lives, leisure activities and sports are important, positive emotions are better remembered than negative ones, freedom of speech matters, birthrates are often high in educated countries, people are rather obese and follow mild sexual norms in developed countries. In societies defined by restraint, people are not that happy and consider that some other force determines their life, leisure activities and sports are given much importance, negative emotions are better remembered than positive ones, freedom of speech does not matter, birthrates are often low in educated countries, people are not obese and follow rather strict sexual norms in developed countries. Moreover, in indulgence cultures, maintaining order is not paramount, while in restraint cultures, order is essential, hence a huge number of policemen are employed. Western Europe and North and South America display features of indulgence cultures, while East European, Muslim and Asian countries restraint ones. In between are Mediterranean cultures that intertwine the two dimensions, indulgence and restraint. 

Although some socio-cultural behavior patterns tend to prevail in a specific culture [46], cultural values that describe two opposite dimensions may co-exist in the same individual and manifest themselves in different situations [47]. In other words, although a specific culture may be characterized broadly by certain values and communication patterns, these may not be defining for each individual that is part of that culture since each person may also have his/her own values that differ from the ones considered representative of that culture [48]. Moreover, even though a low-context communication style is mainly used in individualistic cultures and a high-context communication style in collectivistic ones [49], variations may still occur due to each individual’s values and his/her self-construal [46]. 

Culture, however, is not a static concept, but a dynamic one since societies evolve and their focus changes from security to freedom, as Inglehart contends [50,51,52]. This means that socio-economic evolution brings about security as people no longer feel threatened by external factors that could impact their basic survival needs and, as such, they feel free to develop their expressive self, i.e., their ingenuity and creativity, to choose how to spend their spare time and to develop their moral reasoning and empathic capacity [50,51,52,53,54]. Furthermore, wealth influences national cultural values and their dynamics, particularly in the case of some dimensions such as individualism—collectivism and closeness—power distance [34]. The more developed the country is, the more focused it is on individualistic values and closeness, supporting the emancipation process [34,55]. New technologies also determine cultural change, leading to “partly similar developments in different societies, but there is not the slightest proof that it wipes out variety on other dimensions” [34] (p. 22) since each society has its pre-existing value systems that helps it face new challenges in many different ways.

### 2.2. Overview of Romanians’ Culture-Bound Traits and Values 

Drawing on the extensive international research conducted by other scholars [44,47,56,57,58,59,60,61,62,63,64,65,66,67,68,69,70,71,72,73,74,75] to define the cultural profile of a large number of people, including the Romanian one, D. David [76] critically portrays Romanians and compares them with other nationalities, particularly Americans, indicating both the positive and the negative traits that need to and can be preserved or improved in the future as well as the direction towards which the Romanian people are heading from a cultural viewpoint. Nevertheless, the author emphasizes that the psycho-cultural profile of a country is neither good nor bad unless an ideal psycho-cultural model is accepted, by a specific culture, as a reference standard [76] (p. 164). In other words, the cultural portrait of Romanians drawn by David [76] is inferred from the existing databases, as highlighted by the author himself, outlining only the most important cultural attributes in order to offer some predictive perspectives. 

Thus, Romanian culture is a collectivist one. Yet, it is a moderate collectivism, not an extreme one, caused by the fact that, historically, the country was at the crossroads between various empires, and, as such, people felt always threatened and insecure, feelings that increased during the communist regime. Power, obtained through hard work, is distributed hierarchically, concentrated in a center and highly appreciated by Romanians, which explains the importance given to work and to social status. Power, however, is used in a feminine manner since Romanians try to solve conflicts by compromise and not by violence, which is shown also by a misleading false modesty of the Romanians who wish to have power. Interestingly, although Romania is a moderate-collectivist country, it does not acknowledge leadership properly because many heated arguments occur until an agreement is reached. A typically collectivist lack of trust in unfamiliar people has been extended to familiar ones alike, excepting family members. Romania differs from Western countries in the high importance given to family, in the less significance attributed to friendship, in a less altruistic behavior towards others and in a lower tendency to satisfy their own pleasures. These are intertwined with other cultural values, namely Romanians foster traditions, religious faith and pragmatism as opposed to self-determination and civic spirit, i.e., following the ancestors’ traditions, religious dogmas and self-interest is above deciding for oneself and being a good citizen. Interestingly, although very conformist and religious, but not fundamentalist, Romanians also trust and value technological advancement. All this is linked to the fact that Romanians are highly uncertainty avoidant, which makes them highly emotional and averse to anything that is new and might affect their balance and wellbeing. To better cope with the new, Romanians tend to be conservative, trying to live a life regulated by rules that are, however, sometimes not obeyed. As in the case of the collectivism—individualism dimension, the short-term orientation—long-term orientation dimension records an intermediate score, the balance being slightly tilted towards a long-term orientation. Moreover, femininity is not very much emphasized either and autonomy is higher than in undeniably collectivist societies.

All this points to a culture that is still young, suffering transformations to build its European identity [77]. Thus, David [76] foresees that, as Romania integrates deeper into the European Union, its cultural values will gradually change from collectivism to individualism. This perspective is supported also by the scores Romania recorded in World Values Surveys [58] across time as well as by the results obtained from the research done on the younger generation of Romanians that reveals their individualist and non-collectivist profile [57].

## 3. Materials and Methods

In order to discover whether this wind of change continues to blow also under abnormal conditions, i.e., in pandemic times, we conducted research on the students of Politehnica University of Timișoara (Romania). In other words, research was aimed at identifying the (un)predictibility of the students’ feelings, socio-cultural behavior and communication styles that they employed during the COVID-19 pandemic, sometimes as a means to cope with the new normal. As such, the following research objectives were set: 

RO1. To identify the respondents’ feelings about the first year of the pandemic,

RO2. To identify the respondents’ preferences for a certain design or color of face masks worn during the COVID-19 pandemic,

RO3. To identify the way in which the respondents communicated interpersonally with friends, family members or colleagues during the COVID-19 pandemic,

RO4. To identify the degree of civism indicated by the respondents’ reactions to citizens who were not wearing a face mask when they met.

Although few studies deal with students’ coping behaviors during the COVID-19 pandemic, e.g., students’ coping skills when faced with lockdown fatigue, anxiety, depression, stress and loneliness [78,79,80,81], none of them approaches the topics focused on in the research conducted at Politehnica University of Timișoara (Romania) and presented in this article. As it can be noticed, this research does not intend to draw a complete picture of the Romanian student profile in pandemic times, but it seeks to understand and explain their feelings, coping behaviors and verbal and non-verbal communication in relation to the culture they represent. Hence, RO1 reveals their manner of dealing with the new, RO2 points at their creativity in wearing the face mask, RO3 unveils their communication means and their trust in friends, family members and colleagues, and, finally, RO4 highlights their civism and trust in the authorities, in this case security guards.

### Research Methodology

Data collection was carried out between 1 April and 30 May 2021, during the second semester at the Politehnica University of Timișoara. The following research strategy was resorted to:

- construction of research tools, i.e., a questionnaire that included five opinion questions and five factual questions, for which a 5-point scale (from 1 standing for ‘very little’/‘not at all’ to 5 meaning ‘very much’, where 3 is the mid-point value) was used;
- online distribution of the questionnaire to students at the Politehnica University of Timișoara, Romania, enrolled in on-site programs; for this, a Romanian online survey platform—isondaje.ro—was used;
- building the sample of respondents and a database with their opinions;
- data analysis, with the SPSS program, version 20.00;
- use of a focus group made up of seven students who were employed as co-researchers for the interpretation of the research results. It should be mentioned that this group of students had completed the course “Research Methods in Social Sciences”, and consequently they were aware of the rigor needed while conducting research.

The questionnaire was applied to a sample of 409 subjects, of which 166 men (40.6%) and 243 women (59.4%) participated voluntarily in the survey. The anonymity of the respondents was ensured, and these could drop out of the survey at any time.

Since the analyzed sample consisted only of students, it implies a limitation of the diversity of the population affected by these restrictions. Therefore, even if the sample size was large enough to reach an error margin of ±5%, it would still require multiple samples with greater diversity and a substantially larger number of respondents to make better predictions.

## 4. Research Findings and Discussion

**RO1. To identify the respondents’ feelings about the first year of the pandemic**

The first objective of the study was met by analyzing the following open statement:

*S1. Please describe in three words or three sentences the period of time since the beginning of the pandemic that best expresses your feelings regarding the first year of the pandemic.*

The responses given by the students are summarized in Table 1.

As it can be seen in Table 1, the first year of the pandemic was not very pleasant for the respondents as far as their feelings were concerned. This could be the reason why high percentages for “anxiety, fear, stress” (19.3%), “difficult, hard, tiring, complicated” (19.3%), “isolation, loneliness, quarantine” (14.7%), “monotony, boredom” (11.7%), “sadness” (7.3%), “confusion, chaos, insecurity, uncertainty” (5.1%) were recorded. The so-called positive aspects are reflected in the following answers: “adjustment” (8.6%), “relaxation, relief, freedom, pleasure” (6.1%) and “other choices” (4.9%). Therefore, the responses mainly express the negative aspects of the pandemic that were felt by the respondents. This could be explained by psychological reactance, a term coined by psychologist Brehm in the 1960s, discussing the unpleasant feelings felt by people when prohibited from doing certain things or when sensing that their freedom is being threatened [81].

The students who interpreted the collected data considered that anxiety ranked first for the respondents as the pandemic represented a change in our lives, a fear of the new, and of the unknown. The pandemic period also induced fear and anxiety among the population through the media. Still, after getting used to the imposed restrictions, the population started to feel a relaxation and a relief, partially recovering after the initial shock.

**RO2. To identify the respondents’ preferences for a certain design or color of face mask worn during the COVID-19 pandemic**

The second research objective was achieved by collecting and analyzing the answers to the following question:

*Q1. If you could choose the color or design of face masks worn during the restrictions caused by the spread of the SARS-CoV-2 virus, what would you choose?*

The answers were rated on a 5-point scale and aimed at identifying the students’ preference for a particular color, design or scent of mask, and as such it was intended to reveal their coping strategy with the obligation to wear it during the COVID-19 pandemic (Table 2).

The choice regarding the color of the face mask from the array of possibilities given by the questionnaire showed a relatively interesting grouping. “The colored face mask matching the colors of the clothes I am wearing” (average 3.2) and “the face mask in other/various colors” (average 2.94) ranked first, based on the collected answers. These two categories of answers underline the respondents’ interest in their looks.

The second group of answers were the ones that scored an average between two and three points, i.e., “white face mask” (average 2.75), “blue face mask” (average 2.72), “scented face mask” (average 2.45), “purple face mask” (average 2.26), “face mask in the colors of the establishment I study/work at” (average 2.22), “face mask with different suggestive prints showing my emotional state” (average 2.22). “White” and “blue face masks” were the first two answers as they were colors that could be matched with many types of clothes and were the only ones available in stores in Romania at the beginning of the pandemic. It was only later that the purchasing offer diversified as regards the face masks and their colors. This answer could also be linked to the fact that, according to a recent study [82], people wearing classical surgical masks (usually white or blue) are perceived to be more attractive than people wearing cloth masks. Another study on Japanese women found that pink face masks increase the attractiveness of a woman’s appearance as opposed to a white face mask [83]. The other options depended on the respondents’ various wishes. The “scented” ones were probably required by odor-sensitive people. The respondents choosing “face masks in the colors of the institution they study/work at” were the ones that followed the rules of the establishment in which they carried out their activity, being a means of expressing their loyalty or belonging. There were also the people who liked to showcase the emotional state they were in (happy, sad, etc.) by wearing a face mask expressing it through the printed pattern the face mask displayed.

The last group of answers were those with averages below two, encompassing a variety of options: “green face mask” (average 1.97), “red face mask” (average 1.76), “face mask in the colors of my favorite sports team” (average 1.65), “yellow face mask” (average 1.61), “orange face mask” (average 1.57), “face mask in the colors of the national flag” (average 1.57). The reasons why they ranked last were due to various factors depending on the respondents’ choices. Since orange does not match anything, being a bright color, it ranked second last. The Romanians do not treasure their national flag; therefore, wearing a face mask in the flag’s colors ranked last among the respondents.

The students involved in the research motivated the choice of colors for the face masks using the following statements: “the least I can do is be fashionable with the face mask”; “the face mask has become a part of us”; “if I still have to wear it, I want to look good with it”. Age and desire for social status made the students appreciate the color of the face mask not only based on its usefulness, but also based on color matching. Other students did not take into account the colors and gave answers such as, “I received a blue mask from the city hall and I wore it all the time”. In other words, this category of respondents used the face mask for its usefulness and not as a garment related to fashion.

Another analysis that was carried out was the one discussing face mask color by gender. The analysis of statistical averages based on gender as well as the comparative analysis between the two genders showed statistically significant differences for the following face mask colors: “green face mask”, “blue face mask”, “purple face mask”, “face mask in the colors of the national flag”, “face mask in the colors of the favorite sports team”, “face mask with various imprints”, “face mask matching the clothes”, “scented face mask”, “face mask in various colors” (Table 3). 

The statistical averages of the female color choices of face masks exceeded the averages of the male color choices for the following colors:

- green face mask (average_female_ = 2.08, average_male_ = 1.80), and t = −2.261 (*p* = 0.024);
- blue face mask (average_female_ = 2.84, average_male_ = 2.53), and t = −2.071 (*p* = 0.039);
- purple face mask (average_female_ = 2.55, average_male_ = 1.84), and t = −4.894 (*p* < 0.01);
- face mask with various imprints (average_female_ = 2.47, average_male_ = 1.86), and t = −4.108 (*p* < 0.01);
- face mask matching clothes (average_female_ = 3.58, average_male_ = 2.66), and t = −5.683 (*p* < 0.01);
- scented face mask (average_female_ = 2.63, average_male_ = 2.19), and t = −2.846 (*p* < 0.01);
- face mask in various colors (average_female_ = 3.16, average_male_ = 2.60), and t = −3.581 (*p* < 0.01).

Colors of clothes that are worn, the smell of perfumes as well as the color assortments of clothes are usually a female prerogative, which the responses of this research also underlined.

The statistical averages of the male color choices of the masks exceeded the averages of the female color choices for the following colors:

- face mask in the colors of the national flag (average male = 1.86, average female = 1.37), and t = 4.686 (*p* < 0.01);
- face mask in the colors of a favorite sports team (average male = 2.05, average female = 1.38), and t = 5.972 (*p* < 0.01).

Based on these answers, it can be posited that Romanian men revere the national flag more, being a nation’s symbol and being displayed more in military events, where the male population is more numerous. Also, men are known to be fonder of sports [84,85] from an evolutionary perspective. That is why identifying with a sports team seems to be an extension of a man’s sense of self [86].

**RO3. To identify the way in which the respondents communicated interpersonally with friends, family members or colleagues during the COVID-19 pandemic**

To achieve the third objective, three statements (S1, S2, S3) had to be rated on a scale from 1, i.e., ‘very little’, to 5, i.e., ‘very much’. 

*S1. During the pandemic, meetings with friends took place in various ways. Please go through the following statements and choose the options that best apply to you.*

From the answers presented in Table 4, it can be seen that meetings with friends took place under safe conditions, i.e., outdoors, wearing a face mask (average 3.40), on Zoom/Skype/Whatsapp (average 3.22), indoors, wearing a face mask (average 2.59) or students practically did not meet at all either in the virtual or real world (average 1.68). Among the respondents, there were also some non-conformists who did not wear a face mask no matter where they were with their friends. In this situation, the following answers were recorded: indoors, without wearing a face mask (average 3.04) and outdoors, without wearing a face mask (average 2.81). 

The answers provided by the students participating in the research offered interesting insights into the respondents’ attitudes. Thus, the explanation given for the first answers was that the mask was worn outdoors “because that was the law”. When indoors, the face mask was not worn due to “the absence of a third person to see you” or to “the trust in the person coming to your house, a trust that defeated the virus”. This type of trust was named “the friend-shield effect”, a feeling of safety that appeared in the presence of friends that could subsequently “lower perceptions of infection risk involving crowds outside an immediate social circle” [87].

*S2. During the pandemic, family gatherings took place in various ways. Please go through the following statements and choose the options that best apply to you.*

If, for friendships, wearing a face mask outdoors or meeting online ranked first, for family, as was expected (Table 5), the most important statement proved to be “indoor meetings without wearing a face mask” (average 4.04), while an average of 2.96 was recorded for “outdoors, without wearing a face mask”. Also, for family gatherings, there were many answers indicating compliance with health measures, e.g., “outdoors with a face mask” (average 2.97), “on Zoom/Skype/Whatsapp” (average 2.17), “indoors with a face mask” (average 1, 83) or “not meeting either virtually or face-to-face” (average 1.54).

The explanations given by the students involved in the study were related to the fact that “on holidays or at family events when people met, they did not wear a face mask”; such behavior seeming perfectly normal to them. According to them, this was an expression of “their desire for normality”. That changed when meeting outdoors due to “the legal restrictions”. Moreover, wearing a face mask indoors or outdoors also depended on the special conditions imposed in certain regions due to the level of the infection rate. Therefore, the respondents’ behavior had to obey the rules in force in their hometowns, where they were confined during the pandemic.

*S3. During the pandemic, meetings with colleagues took place in various ways. Please go through the following statements and choose the options that best apply to you.*

Meetings with colleagues during the pandemic were safer from a health perspective, as presented in Table 6. Thus, in the first three places, the following answers could be found, i.e., “on Zoom/Skype/Whatsapp” (average 3.50), “outdoors, wearing a face mask” (average 2.60) and “indoors, wearing a face mask” (average 2.23). The less hygienic encounters were “indoors, without a face mask” (average 2.05). The cases of “non-encounters in either the virtual or the real world” had an average of 2.02 while the “outdoor meetings, without wearing a face mask” (average 2) ranked last.

The explanations given by the students for these statements were that “the respondent students had online classes” and did not meet, anyway. Most of them met on Zoom, “not living in the same region/town/city”. “The answers involving meetings with a face mask dominate as you cannot trust someone you do not see”. In other words, online activities and distrust of strangers made people more obedient where health restrictions were concerned.

**RO4. To identify the degree of civism indicated by the respondents’ reactions to the citizens who were not wearing a face mask when they met.**

To meet the last research objective, only one question (Q1) was asked that envisioned a possible situation to which the respondents could react in five ways, each being rated on a 5-point scale (Table 7). 

*Q1. If, in the middle of a pandemic, you saw a person not wearing a face mask at a shopping center, what would you do?*

The question the respondents had to answer was a rather delicate one, but of civic importance. It seemed that quite a few respondents “would do nothing” (average 3.14) while another category “would do something, even if not knowing exactly what that would be” (average 2.28), meaning they would act impulsively. Civic answers were the choice of many respondents, i.e., “I would reprimand that person” (average 2), “I would request an explanation from that person” (average 1.94) or “I would call the security guard” (average 1.87).

The explanations given by the students were interesting and humane. According to them, some respondents “would not do anything” because they probably did the same thing many times, i.e., they did not always wear a face mask for various reasons. Other explanations that were given were related to the belief that “only the person who is not wearing the face mask is affected by viruses, not those around them”. Others did not interfere because they feared negative consequences such as: “I do not express my opinions because we live in a crazy world in which people do not want to start such discussions”, “an unnecessary quarrel might arise”. Then there is the issue of “disagreements regarding the wearing of face masks”.

## 5. Conclusions

As far as the first research objective was concerned, the respondents’ feelings during the pandemic were rather negative, a fact shown not only by the respondents’ answers, but also by the interpretations given by the students involved in the research. Moreover, the stress felt during this period was generated by the fact that an infection with SARS-CoV-2 virus might be lethal. The news provided by the media had an anxiogenic effect by presenting the viral effects of this highly publicized virus. The fear also arose due to the fact that, through the media, a large part of the population was informed, which was a positive aspect, but it also led to panic through images, news or alarming debates, a clear negative aspect. In other words, the cultural trait of the Romanians showing their high emotionality when facing uncertainty and the new [76] was present also in the younger generation and was largely exploited and even intensified by the mass-media. In this context, the activity of the mass-media should be better regulated and stricter rules should be imposed in Romania in order to avoid creating panic in a population that is already prone to feeling stressed and desperate whenever an uncertain future looms. 

The second research objective sought to observe whether the restrictions imposed by the pandemic were also expressed in the choice of colors, design and scent of face masks. From the collected answers, two types of trends could be identified, i.e., a rather conformist one, in which people had to wear the face mask for health reasons and its color has no importance whatsoever, and a second trend, specific to people who wore the face mask and tried to integrate it to their outfit as far as style and colors were concerned. This second trend points to a rather unexpected result since, although Romanians are moderately collectivist [76,88], the younger generation tends to be creative in their daily life and to place more emphasis on the appearance at least in terms of fashion. Hence, creativity and artistic sense could be used to help the younger Romanian population better face stressful and constraining situations as well as to implement unpopular but useful measures in exceptional times, such as the COVID-19 pandemic.

As for the third research objective, meetings with friends took place safely, indoors or outdoors with a face mask, or online. There were also exceptions, i.e., face-to-face meetings, indoors or outdoors, without a face mask, based on the trust and comfort that a friendship offers, studies suggesting that love has the potential to promote health and reduce stress [89]. Family gatherings were a source of normality for all the respondents, and this manifested in opting for indoor meetings without a face mask. Family events or gatherings were more common, being an oasis of normality that people needed, this behavior intended to be as natural as possible. The highest percentage was scored by meetings with colleagues that complied with the sanitary restrictions. Even though for many of the respondents the activities took place online during the pandemic, short returns to the university happened under safety and hygienic conditions. The respondents’ behavior regarding the adoption of health measures depended very much on the degree of closeness between the interlocutors, as well as on the level of infection in the respondents’ region/place of residence. The results, in this case, are consistent with other cultural studies on Romanians [76] that show that people trust neither familiar nor unfamiliar persons, but family members. Therefore, only amidst family did the younger generation feel safe not to wear a face mask. Furthermore, the results showed a high degree of conformism in obeying the rules which is typical of highly avoidant societies such as the Romanian one [34,43,44,45,76].

The fourth research objective, setting out to identify the degree of civism regarding the wearing of face masks in public places, showed the respondents’ non-involvement in the public issue of all citizens wearing a face mask, which could have multiple explanations. The interviewed students’ opinions on this matter were mainly related to the fact that the respondents likely also had moments of non-compliance with the sanitary regulations and, therefore, they felt guilty for reprimanding others of non-compliance with the hygiene rules. Another opinion referred to people’s desire not to generate potential conflicts. The behavior revealed by the respondents’ answers emphasizes that even young Romanians sometimes bend the rules and avoid conflicts by reaching a compromise, which is, in fact, a feminine manner of dealing with power distance. In this case, conflict was avoided by not adopting a civic attitude and, as such, by not taking action when rules were disobeyed, and even by not disclosing the incident to the authorities, which reflects the collectivist lack of trust in public institutions [34,43,44,45,76,90]. 

The above-mentioned research objectives may open new paths of discussion and research on the topics of culture and of people’s reactions to unexpected and possibly life-threatening situations. Moreover, during the COVID-19 pandemic, people’s behavior in situations other than the ones approached in the research presented above could be analyzed from a cultural perspective in order to learn lessons that could help the authorities better cope with crisis situations. 

## Figures and Tables

**Table 1:** Description of the first year of the pandemic by the respondents.

Description of the Feelings Experienced during the First Year of the Pandemic	Frequency	Percentage
Anxiety, fear, stress	79	19.3
Difficult, hard, tiring, complicated	79	19.3
Isolation, loneliness, quarantine	60	14.7
Monotony, boredom	48	11.7
Adjustment	35	8.6
Sadness	30	7.3
Relaxation, relief, freedom, pleasure	25	6.1
Confusion, chaos, insecurity, uncertainty	21	5.1
Other choices	20	4.9
Pensive, self-reflection period	12	2.9
Total	409	100.0

**Table 2:** Averages for the respondents’ face mask choices.

Choices Made for Wearing Face Masks	Average
1. Colored face mask matching the colors of the clothes I am wearing	3.2
2. Face mask in other/various colors	2.94
3. White face mask	2.75
4. Blue face mask	2.72
5. Scented face mask	2.45
6. Purple face mask	2.26
7. Face mask in the colors of the establishment I study/work at	2.22
8. Face mask with different suggestive prints showing my emotional state	2.22
9. Green face mask	1.97
10. Red face mask	1.76
11. Face mask in the colors of my favorite football team	1.65
12. Yellow face mask	1.61
13. Orange face mask	1.57
14. Face mask in the colors of the national flag	1.57

**Table 3:** Face mask color by gender.

Face Mask Color	Gender	N	Mean
Green face mask	male	166	1.80
	female	243	2.08
Blue face mask	male	166	2.53
	female	243	2.84
Purple face mask	male	166	1.84
	female	243	2.55
Face mask in the colors of the national flag	male	166	1.86
	female	243	1.37
Face mask in the colors of favorite sports team	male	166	2.05
	female	243	1.38
Face mask with various imprints	male	166	1.86
	female	243	2.47
Face mask matching clothes	male	166	2.66
	female	243	3.58
Scented face mask	male	166	2.19
	female	243	2.63
Face mask in various colors	male	166	2.60
	female	243	3.16

**Table 4:** Statements regarding meetings with friends during the pandemic.

Statements Regarding Meetings with Friends during the Pandemic	Averages
1. Outdoors, wearing a face mask	3.40
2. On Zoom/Skype/Whatsapp, etc.	3.22
3. Indoors, without wearing a face mask	3.04
4. Outdoors, without wearing a face mask	2.81
5. Indoors, wearing a face mask	2.59
6. We have not met either virtually or face-to-face	1.68

**Table 5:** Statements regarding family gatherings during the pandemic.

Statements Regarding Family Gatherings during the Pandemic	Average
1. Indoors, without wearing a face mask	4.04
2. Outdoors, wearing a face mask	2.97
3. Outdoors, without wearing a face mask	2.96
4. On Zoom/Skype/Whatsapp, etc.	2.17
5. Indoors, wearing a face mask	1.83
6. We have not met either virtually or face-to-face	1.54

**Table 6:** Statements regarding meetings with colleagues during the pandemic.

Statements Regarding Meetings with Colleagues during the Pandemic	Average
1. On Zoom/Skype/Whatsapp, etc.	3.50
2. Outdoors, wearing a face mask	2.60
3. Indoors, wearing a face mask	2.23
4. Indoors, without wearing a face mask	2.05
5. We have not met either virtually or face-to-face	2.02
6. Outdoors, without wearing a face mask	2.00

**Table 7:** Respondents’ reactions to citizens not wearing a face mask in a shopping center.

Possible Reactions	Average
1. I would not do anything	3.14
2. I would do something, but I do not know what	2.28
3. I would reprimand that person	2.00
4. I would request an explanation from that person	1.94
5. I would call the security guard	1.87

## Data Availability

The data is available upon request.
